# Evaluation of the birth plan implementation: a parallel convergent mixed study

**DOI:** 10.1186/s12978-020-00989-6

**Published:** 2020-09-07

**Authors:** Parivash Ahmadpour, Sanaz Mosavi, Sakineh Mohammad-Alizadeh-Charandabi, Shayesteh Jahanfar, Mojgan Mirghafourvand

**Affiliations:** 1 Department of Midwifery, Faculty of Nursing and Midwifery, Tabriz University of Medical Sciences, Tabriz, Iran; 2 Women Reproductive Health Research Center, Tabriz University of Medical Sciences, Tabriz, Iran; 3 Public Health Department, Central Michigan University, Mount Pleasant, MI USA; 4 Social determinants of Health Research Center, Tabriz University of Medical Sciences, Tabriz, Iran

**Keywords:** Birth plan, Birth experience, Mixed-method

## Abstract

**Background:**

Pregnancy, birth, and motherhood are among the most important events of every woman’s life. Training and participation of mothers in the decision-making process of delivery play an essential role in physical as well as psychosocial preparation of the mother. The healthcare system can improve and enhance the level of care by involving the patient in their self-care process. The aim of the present study is to assess the implementation of the birth plan for the first time in Iran in Tabriz city.

**Methods/design:**

The present study uses a mixed-method with a parallel convergence approach, including both quantitative and qualitative phases. The quantitative phase is a randomized controlled clinical trial performed on 106 pregnant women, 32–36 weeks of pregnancy, referring to Taleghani educational hospital in Tabriz city. The participants will be assigned into intervention and control groups using a randomized block method. A training session will be held about the items of the birth plan checklist at weeks 32–36 of gestation for the participants in the intervention group, whereby a mother-requested birth plan will be developed. It will then be implemented by the researcher after admitting them to the delivery ward. Also, those in the control group will receive routine care. During and after the delivery, the questionnaire of delivery information, neonatal information, and Delivery Fear Scale (DFS) will be completed. Also, a partogram will be completed for all participants by the researcher. The participants in both groups will be followed up until six weeks post-delivery, whereby the instruments of Childbirth Experience Questionnaire (CEQ2.0), Edinburgh’s Postpartum Depression Scale and PTSD Symptom Scale 1 (PSS-I) will be completed six weeks 4–6 weeks postpartum by the researcher through an interview with participants in Taleghani educational hospital. The general linear model and multivariate logistic regression model will be used while controlling the possible confounding variables.

The qualitative phase will be performed to explore the women’s perception of the effect of the birth plan on childbirth experience within 4–6 weeks postpartum. The sampling will be of a purposeful type on the women who would receive the birth plan and will continue until data saturation. In-depth, semi-structured individual interviews would be used for data collection. The data analysis will be done through content analysis with a conventional approach. The results of the quantitative and qualitative phases will be analyzed separately, and then combined in the interpretation stage.

**Discussion:**

By investigating the effect of implementing the birth plan on the childbirth experience of women as well as other maternal and neonatal outcomes, an evidence-based insight can be offered using a culturally sensitive approach. The presentation of the results obtained from this study using the mixed method may be effective in improving the quality of care provided for women during labor.

**Trial registration:**

Iranian Registry of Clinical Trials (IRCT): IRCT20120718010324N58. Date of registration: July 7, 2020. URL: https://en.irct.ir/user/trial/47007/view

## Plain English summary

Women’s choice and control impact on birth experiences. Women’s positive and negative recollections of their birth experiences are related more to feelings and exertion of choice and control than to specific details of the birth experience, birth plan affecting her recollection of the birth experience. The usage of a birth plan is common in developed countries, but it is rather new in developing countries. So far, in Iran, no birth plan has been established. The birth plan can be an effective and important tool to achieve physiological delivery, better control the process of labor and delivery, and enhance satisfaction with childbirth. The present study uses a mixed-method with a parallel convergence approach, including both quantitative and qualitative phases. The present research has two phases: 1) quantitative, which is randomized controlled clinical trial, 2) qualitative with the aim of exploring the women’s perception about the effect of the birth plan on their childbirth experience. The results of the quantitative and qualitative phases will be analyzed separately, and the results will be presented jointly. By implementing the birth plan, the advantages and obstacles of the birth plan in Iranian society can be clarified. In case of observing the positive effect of this plan, its protocol can be provided to planners and policymakers so that they could use it to improve the experience of Iranian women and to enhance maternal and neonatal health outcomes and to increase the active participation of women in the labor and delivery process.

## Background

The birth plan was first put forward in the 1980s in Europe and US in response to the approach of increasing “medicalization of labor” with the aim of improving women’s health, reducing adverse maternal outcomes as well as refining the communication between women and healthcare professionals [1, 2]. The presumption of the birth plan was that it would allow women to express their expectations and needs about the process of childbirth. This plan was a huge movement for reclaiming women’s rights and patient’s reproductive rights. The birth plan has been a communication tool written by pregnant women [3], including their preferences for managing their labor and delivery. It provides different ways for discussion between the care provider and pregnant woman and helps her to achieve better experience of delivery through greater control over their labor. The birth plan is written by women in consultation with the healthcare team; just like the written informed consent sheet, informed and freely chosen decisions should be respected even if they are presented verbally [4, 5].

The WHO recommends the use of birth plan and mostly emphasizes normal processes without interventions [6]. Nevertheless, the birth plan should be beyond a checklist reflecting the preferences and emotions as well as understanding of women about the physiology of birth and women’s need for safety and support [1]. Support and communication during labor enhance the level of satisfaction with the delivery, and this effective communication should begin from the time of admission and constantly continue during labor; when not all things proceed as planned, the information should be actively given in women’s decision-making process [7]. Consequently, the extent of satisfaction with delivery in women grows with the birth plan and improves their delivery experience [8].

The childbirth experience is an important psychological phenomenon postdelivery whose psychological effects lead to various short-term and long-term adverse health outcomes, with the negative experiences of childbirth being well associated with Post Traumatic Stress Disorder (PTSD) and depression [9–12]. The childbirth experienced is stated to be considered a harmful event for some women [13]. PTSD postdelivery is related to the psychological torments of labor. Generally, 5–20% of women may report delivery-associated traumatic events [14]. According to a systematic review, this prevalence of Post-traumatic stress disorder has been reported to be 25% among Iranian women [15]. Research has shown that the childbirth experience may be affected by a wide range of provisions, including support, external control, internal locus of control, as well as obstetrical complications [13–16].

The practice of a birth plan is common in developed countries, but it is rather new in developing countries [17]. So far, in Iran, no birth plan has been established. The birth plan can be an effective and important practice to achieve physiological delivery, improve communication with the healthcare staff, control the process of labor and delivery, improve the maternal and neonatal outcomes, and enhance satisfaction with childbirth. Indeed, the key to achieving these goals is increasing the degree of realization of the birth plan [16–18]. Hence, some policies are required to encourage the use of a birth plan along with executive improvement and adaptation of this plan. It seems that assessment of the effect of implementing a birth plan using a mixed-method approach would help in better understanding the issue. Furthermore, one of the advantages of the birth plan is to decline the cesarean section rate considering the increasing rate of cesarean delivery in Iran [19]. Also, it is expected that a birth plan may support current population policies in Iran [20] by improving neonatal and maternal outcomes. Because, the birth plan establishes a better and easier birth experience for women, leading to adopting vaginal birth over the selective cesarean section. Also, in order for the program to succeed, the healthcare providers should be trained about evidence-based birth care causing the promotion of physiological delivery and developing skills in women to achieve drug-free and intervention-free delivery. Possibly, the implementation of the birth plan could enhance childbirth satisfaction among women.

### Study aim

To assess the effectiveness of the birth plan in women of Tabriz-Iran.

### The specific objectives of the quantitative phase are

1. Comparing the mean score of childbirth experience in the studied groups (having a birth plan, not having a birth plan)
2. Comparing the mean duration of the active phase of delivery in the studied groups (having a birth plan, not having a birth plan)

### The secondary objectives of the quantitative phase are

1. Comparing the mean duration of the second stage of delivery in the studied groups (having a birth plan, not having a birth plan)
2. Comparing the mean duration of the third stage of delivery in the studied groups (having a birth plan, not having a birth plan)
3. Comparing the mean score of fear of delivery in the studied groups (having a birth plan, not having a birth plan) by controlling the baseline score
4. Comparing the frequency of vaginal delivery in the studied groups (having a birth plan, not having a birth plan)
5. Comparing the mean PTSD score in the studied groups (having a birth plan, not having a birth plan)
6. Comparing the frequency of neonatal hospitalization in NICU in the studied groups (having a birth plan, not having a birth plan)
7. Comparing the mean postpartum depression score in the studied groups (having a birth plan, not having a birth plan) by controlling the preintervention depression score
8. Comparing the mean neonatal Apgar score in the first and fifth minutes in the studied groups (having a birth plan, not having a birth plan)

### The specific objective of the qualitative phase is

Exploring the mothers’ perception of the effect of implementing the birth plan on their childbirth experiences

## Methods/ design

### Study design

**Fig. 1:**
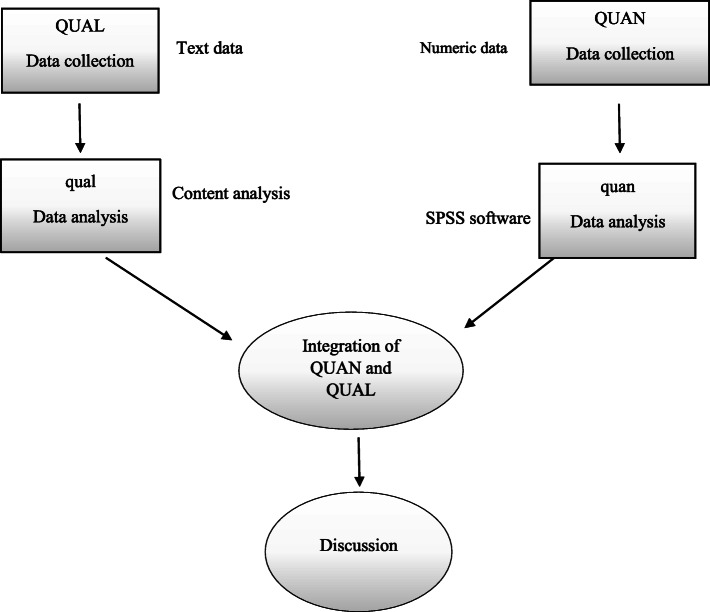
Study visual diagram

The present research is a convergent parallel mixed study with a pragmatic paradigm. We will be able to enrich our findings if we assess the effect of the birth plan through both qualitative and quantitative studies. Therefore, current research has two phases: 1) quantitative, which is a randomized controlled clinical trial, 2) qualitative. The findings of the quantitative and qualitative phases will be analyzed separately, and the final interpretation would benefit from both evidence obtained from both study phases (Fig. 1).

### Quantitative study

#### Study design

The quantitative phase of the study is a parallel randomized controlled clinical trial. The target population is pregnant women with a gestational age of 32–36 weeks referring to the obstetrics clinic of Taleghani educational hospital, Tabriz, Iran.

#### Inclusion criteria

Minimum age of 18 years, residence in Tabriz city, willingness to undergo vaginal delivery, gestational age of 32–36 weeks, depression score lower than 13 based on EPDS, singleton pregnancy, literacy [reading and writing in Persian], planning for delivery in Taleghani hospital, women with the first and second delivery.

#### Exclusion criteria

Non-cephalic presentation, twins/triplets, having indications for C-section including abnormal presentation, obstetrics problems including placenta previa, post-cesarean vaginal delivery, placental abruption, preeclampsia, high-risk pregnancies such as diabetes, cardiovascular disease, etc. stillbirth, or abnormal fetus.

#### Sample size

The sample size in this study was calculated based on the two variables of “childbirth experience” and “duration of the active phase of labor” using G-power software. Based on the results of the study by Ghanbari Homaei et al. [21] on the variables of childbirth experience and considering m_1_ = 2.71, m_2_ = 3.25 (assuming 20% increase in response to the intervention), sd_1_ = sd_2_ = 0.73, one-sided α = 0.05, and power = 90%, the sample size was calculated 32 participants; considering 10% attrition, the sample size will be 35 in each group. According to the variable of the duration of the active phase of labor and concerning m_1_ = 276.7, m_2_ = 221.4 (assuming 20% reduction in response to intervention), sd_1_ = sd_2_ = 91.3, α = 0.05, and power = 90%, it was calculated as 48 participants, and eventually, it will be 53 as the sample size in each group, given 10% possible attrition. Since the sample size calculated based on the variable of the duration of the active phase of labor would be more extensive. Thus the final sample size was considered 53 in each group.

#### Sampling

To collect the quantitative data, the researcher would refer to the obstetrics clinic of the Taleghani hospital and would choose the eligible women referring to this clinic through a convenient sampling method. Then, a comprehensive description of the study will be given to the women about the research, its goals, and its methodology, and if they were willing to participate, a written informed consent form would be signed. Next, questionnaires would be completed about the socio-demographic and obstetrical history followed by filling up the Edinburgh’s Postpartum Depression Scale (EPDS) via interview. Note that pregnant women with a depression score of less than 13 will be included.

#### Randomization and allocation concealment

To allocate the participants to the studied groups, a randomized block method with 4- and 6-blocks will be used with an allocation ratio of 1:1. For allocation concealment, the type of intervention is written in a piece of paper and will be placed inside consecutively numbered opaque envelopes. Random allocation and allocation concealment will be conducted by a person not involving in the sampling and data collection. After the study participants signed the consent form, the relevant envelope will be opened, and the intervention will be implemented.

#### Intervention and follow-up

After allocating the participants in this study groups, for those in the intervention group, an educational session will be held to explain the birth plan, whereby all items of the birth plan will be explained. Also, an educational pamphlet in which all items of the birth plan checklist are explained will be provided to the participants of the intervention group. During the session, the researcher’s phone number will be provided to the mothers, and she will answer any questions of participants about the birth plan. In the case of the mother’s preparation, the mother-requested birth plan will be developed by the researcher in the very first session. If the mothers are not prepared, however, the next session will be set up with the participant to develop the birth plan. The established birth plan will be confirmed by an Obstetrician. Within the interval between the in-person session and time of delivery, the researcher will be in contact with mothers through phone, or in case the mother wished, will contact in person. Also, the participant will be asked to contact the researcher as soon as they referred to the hospital. With the entrance of the participants to the labor ward of the hospital, the birth plan will be implemented by the researcher. Also, the participants in the study groups will receive routine and standard care. Immediately during and after delivery, the delivery information questionnaire, neonatal information, and Delivery Fear Scale (DFS) will be completed. Also, a partogram will be completed by the researcher for all participants. The participants in both groups will be followed-up for 6 weeks postpartum. The childbirth experience questionnaire-2 (CEQ 2.0), EPDS, and PTSD Symptom Scale 1 (PSS-I) will be completed 6 weeks after delivery in Taleghani Hospital by the researcher through interviews.

### Quantitative study

#### Scales and data collection

To collect quantitative data, socio-demographic as well as obstetrics history, birth plan checklist, Childbirth Experience Questionnaire (CEQ 2.0), Edinburgh’s Postpartum Depression Scale (EPDS), partogram, Delivery Fear Scale (DFS), PTSD Symptom Scale 1 (PSS-I), and a checklist of maternal as well as neonatal outcomes will be used, and the information will be collected through face-to-face interview method.

##### Socio-demographic and obstetric characteristics questionnaires

This questionnaire will contain items about age, level of education, occupation, having companion, religion, ethnicity, marital status, residence status, household income, being rural or urban, etc. The obstetric questionnaire has items about the number of pregnancies, number of deliveries, number of abortions, history of infertility, etc.

##### Childbirth experience questionnaire (CEQ 2.0)

This instrument contained 25 items and measures the childbirth experience for women. The questionnaire covers the following areas: personal capacity (locus of control is a psychological concept that refers to how strongly people believe they have control over the situations and experiences that affect their lives.], personal feelings about childbirth and labor pain), professional support (midwifery care and information), perceived security (sense of security and memories of childbirth), and participation (the person’s ability to change the position, movements, and pain mitigation during labor). Specifically, 23 items are multiple-choice (with four options), and three items are completed based on a visual analog scale (VAS). The responses are in the form of absolutely agree (score 1), often agree (score 2), often disagree (score 3), and absolutely disagree (score 4). The items responded based on VAS will be changed into values 1–4: scores 0–40 (score 1), scores 41–60 (score 2), scores 61–80 (score 3), and scores (81–100) (score 4). Sentences with negative concepts (experience of severe pain, sense of fatigue, fear, and having bad memory) are scored negatively. High mean values in this instrument represent a more positive childbirth experience [22]. Also, the reliability and validity of the Persian version of this questionnaire have been determined in the research setting by Ghanbari Homaei et al. [23]. Where the Cronbach alpha coefficient of items and the intra-class correlation coefficient was reported to be 0.93 and 0.97, respectively [23], this instrument will be completed 6 weeks postpartum via interview.

##### Edinburgh’s postpartum depression scale (EPDS)

This questionnaire is used to measure postnatal depression as well as depression during pregnancy, first developed by Cox et al. in 1987. This instrument consists of 10 multiple-choice questions (with four options). In some items, the choices are ordered from low to high (items 1, 2, and 4), while in other cases from high to low (items 3, 5, 6, 7, 8, 9, 10). The options for each item claim a score from 0 to 3 based on the severity of symptoms. The score gained by the person is obtained through summing up the scores of the ten items, which can vary from 0 to 30. This questionnaire will be completed through an interview by the researcher when the mother refers to routine checkups during 32–36 weeks of gestation. The mothers acquiring scores above the threshold limit of 12 have depression with different severity. Also, 6 weeks into postpartum, we will complete the questionnaire again via an interview to examine postnatal depression. The validity of this scale using the method of determining the concurrent correlation coefficient was calculated to be 0.78. Also, the reliability using Cronbach alpha method and split-half method was estimated as 0.75 [24]. Montazeri et al. reported the Cronbach alpha value associated with the postnatal period as 0.77, with the intra-class correlation coefficient of 0.80 [25].

##### Delivery fear scale (DFS)

In order to assess the fear, the delivery fears scale will be used, first designed by Wijma. DFS is a valid 10-item self-assessment questionnaire, capturing the fear of delivery during labor through scores ranging from 1 = absolutely disagree to 10 = absolutely agree. DFS is a questionnaire that can be completed almost effortlessly within 60–90 s at any time of labor. Higher scores represent greater fear [26]. The Persian version of DFS is a reliable and valid tool to measure fear in the delivery room in the active phase of labor [3–5]. Cronbach’s alpha of this questionnaire in the study by Irvani et al. has been calculated 0.77, which is going to be published.

In order to assess the fear of childbirth during pregnancy, fear of delivery scale (W-DEQ-Version A) will be used. This questionnaire, first designed by Wijma et al. in 1998, measures fears and expectations related to predelivery with 33 items. The mothers express their personal emotions and perceptions based on a 6-point Likert scale (zero = never, 5 = many times). Generally, the total score is obtained by summing up the score of all items, with scores ranging between 0 and 165. The score of 100 is considered the cutoff point, with higher scores showing a higher score of fear of delivery. Items 2 and 3 are reverse-scored. Wijma et al. estimated the reliability of the questionnaire through the split-half method and the Cronbach alpha coefficient as 0.89 and 0.92, respectively. Scores equal to or less than 37, 38–65, 66–84, and greater than 85 represent low, moderate, high, and intense fear [27]. The reliability and validity of this questionnaire have been determined by Mortazavi et al. in Iran, with a Cronbach alpha coefficient of 0.91 [28].

##### Birth plan checklist

The idea of the birth plan was put forward by Kitzinger in the US in the 1980s [29]. The birth plan was rapidly executed in some European countries and is used in 78% of delivery rooms in England. This includes women’s preferences in labor, mobility, monitoring, pain relief, pharmacological options, acceleration of labor, pushing, respiration and care of the child, and cesarean. It is completed during pregnancy in consultation with healthcare staff or obstetricians by pregnant women [4].

##### PTSD symptom scale 1 (PSS-I)

It includes 17 items that completely cover all criteria of the fourth version of the diagnostic and statistical manual of psychiatric disorders (DSM-IV) to diagnose post-traumatic stress disorder and marks the severity of symptoms of every criterion using a Likert scale. The indicators of this disorder include symptoms associated with re-experiencing (four items), symptoms related to avoidance (seven items), and symptoms pertain to arousal (6 items). In the case of having one or more symptoms of re-experiencing and three or more symptoms related to avoidance, and two or more factors related to arousal, PTSD diagnosis is made. The range of scores is 0–51. The Cronbach alpha of the Persian version of the tool is 0.88, and the Kappa coefficient calculated three test-retest methods has been reported as 1. This questionnaire is completed 4–6 weeks postpartum [30].

##### Partogram form

Partogram is a simple, inexpensive, and valid diagram, which is indeed the best instrument for monitoring the process of delivery as well as maternal and neonatal health. It allows healthcare staff to visually express the details of delivery. It involves registering information regarding the maternal health status, fetal health status, recording the process of delivery, and managing the delivery. Indeed, it is an early warning system that remarkably helps in decision-making on the timely referral of the mother [31].

The reliability and validity of the CEQ 2.0 and EPDS have already been determined and confirmed in the research setting of this plan (Tabriz city). To determine the validity of the birth plan checklist, we will use the translation, content, and face validity of the questionnaire. Specifically, the questionnaires will be filled by ten specialists, the head of the Taleghani Hospital, and 20 mothers. After collecting their opinions, the necessary corrections will be made to the checklist based on the feedback acquired. Also, the reliability of the other questionnaires, including DFS and PSS-I, will be determined through the test-retest method on 20 participants with a two-week interval as well as through specifying the Cronbach alpha coefficient and intra-class correlation coefficient (ICC).

### Data analysis

The obtained information at this phase will be analyzed by SPSS 22. To describe the socio-demographic and obstetrics characteristics, descriptive statistics, including frequency (percentage), mean (SD), in case of normality, will be used, while the median (percentile 25 to 75) will be applied for abnormal data. To compare the variables of the childbirth experience, postnatal depression, post-traumatic symptoms, as well as maternal and neonatal outcomes across the studied groups, in bivariate analysis, one-way analysis of variance and chi-square tests will be used; in multivariate analysis, multivariate linear regression or multivariate logistic regression will be employed by controlling the socio-demographic, obstetrics and delivery characteristics.

### Qualitative study

#### Study design

The method of the qualitative phase of the study is a qualitative content analysis with a conventional approach. The main advantage of this approach is obtaining direct information from the study without imposing predetermined issues or theories [32].

#### Data collection

To collect qualitative data, within 4–6 weeks postpartum, in-depth, semi-structured individual interviews with open questions will be used. The sampling method of the qualitative section is purposeful and will have the maximum variety in terms of demographics. Those who are able to express themselves and willing to participate in the study will be selected deliberately. Before implementing the qualitative stage, the desired questions will be designed in light of interview guidelines in cooperation with the research team. The methods of acquiring valid data and the procedure of focusing on research questions will be reviewed with the research team members, and the interview will begin with predetermined questions. Also, in the course of the interview, deepening and exploratory questions will be presented based on the type of response to each question to explore the depth of women’s experience. The examples include, “what do you mean? “, “Why is that?”, “Please explain more”, “would you please make an example so that I can understand what you mean”. During the interview, the researcher will record nonverbal data such as tone of voice, manifestations of the face, and position of participants in a particular sheet while mentioning the time and place of the interview. The sampling will be performed from among the individuals in the birth plan group as purposeful and will continue until data saturation, i.e., when no new information or code is obtained.

#### Data analysis

In this method, data analysis begins with reading all texts repeatedly, such that the researcher is immersed in the data and gains a general impression. Next, the texts are read word by word for extracting codes, and the first objective words of the text which seem to cover the main concepts or thoughts are identified. Next, the researcher proceeds through the text by taking notes from the initial analysis as well as her primary beliefs and thoughts. As the process goes on, the labels of codes that reflect more than one main thought will emerge. They are often extracted directly from the text. Then, the codes are categorized based on differences and their relationship with each other. The created subcategories are used for organizing and grouping main categories [33, 34].

### Ethics approval and consent to participate

This study has been approved by the ethics committee of Tabriz University of medical sciences (ethics code: IR.TBZMED.REC.1399.278). The quantitative phase of the study (randomized controlled clinical trial) has been registered in the Iranian Clinical Trial Registration Center (code: IRCT20120718010324N58). Inform written consent will be received from all participants in both quantitative and qualitative status. The participants will be assured about the confidentiality of information and privacy of their identity. It will also be explained that they are allowed to quit the study at any stage of intervention, and their refusal to cooperate is free at any stage, and no change will be made in presenting or in the quality of services offered to them.

## Discussion

Pregnancy, childbirth, and maternity are among the most important events of women’s lives, which are a complex and important experience for women, with long-term effects throughout their lives. This experience affects the welfare and future of women, their relationship with their child, as well as their relationship with their partner. If childbirth conditions are stressful for women, they may become fragile and vulnerable in their reproductive period [12, 35]. The participation of mothers in the decision-making process plays a significant role in managing stress and maternal support. Also, the training and participation of mothers in the decision-making process during delivery plays an important role in physical as well as psychosocial preparation of the expectant mother [36].

Healthcare staff is in a unique situation to provide information, training, and support for women and their families [37]. The healthcare system can improve and enhance the level of care by involving the patient in their self-care process [38]. Over the past few decades, the values, preferences, and desires of patients have been gaining more attention and services. These are mostly oriented to enhancing the level of care as well as patient-centered care [4]. Participation in decision-making is a method in which the therapist and patient both participate in the process of treatment together using the best scientific evidence available. The birth plan is indeed involving women in decisions on the labor process and delivery [39].

The importance of the birth plan originates from the principle of respecting biology and independence, thus causing enhanced control of women over the delivery process as well as their satisfaction [38]. Also, due to presenting information and creating awareness and communication with women, it can reduce their fear and create positive feedback for women [16–19]. Many birth plans include a checklist with a small guideline to select intervention as well as the reasons for selecting intervention in labor and delivery [12, 20]. The birth plan facilitates communication with obstetricians and midwives, causing satisfaction of women with the delivery process and enhancing participation in the decision-making process for their delivery [39].

This proposal has several strong points. It will fill the important gaps of knowledge in the support and participation of women during labor and delivery using the birth plan in an Iranian setting. Thus, it will expectedly have important clinical outcomes. Since so far no study has been performed in Iran regarding the effect of birth plan in Iran, thus this study has been designed with a mixed approach, so that through integrating different approaches and methods, a deeper understanding of concepts could be gained to support the birth plan.

By implementing the birth plan, the advantages and obstacles of the birth plan in Iranian society can be clarified. In case of observing the positive effect of this plan, its protocol can be provided to planners and policymakers so that they could use it to improve the experience of Iranian women and to enhance maternal and neonatal health outcomes and to increase the active participation of women in the labor and delivery process.

## Data Availability

Not applicable.

## Abbreviations

CEQ 2.0: Childbirth experience questionnaire 2.0
DFS: Delivery fear scale DFS
PTSD: Post traumatic stress disorder
WHO: World health organization
VAS: Visual analog scale
ICC: Intra-correlation coefficient
DSM-IV: Diagnostic and statistical manual of mental disorders 4th edition
SD: Standard deviation

## References

[1] Lothian J (2006). Birth plans: the good, the bad, and the future. J Obstet Gynecol Neonatal Nurs.

[2] Aragon M, Chhoa E, Dayan R, Kluftinger A, Lohn Z, Buhler K (2013). Perspectives of expectant women and health care providers on birth plans. J Obstet Gynaecol Can.

[3] Hildingsson I, Johansson M, Karlström A, Fenwick J (2013). Factors associated with a positive birth experience: an exploration of swedish women's experiences. Int J Childbirth.

[4] Bailey JM, Crane P, Nugent CE (2008). Childbirth education and birth plans. Obstet Gynecol Clin N Am.

[5] Hollins Martin CJ (2008). Birth planning for midwives and mothers. Br J Midwifery.

[6] Technical Working Group WHO (1997). Care in normal birth: a practical guide. Birth.

[7] Anderson CJ, Kilpatrick C (2012). Supporting patients' birth plans: theories, strategies & implications for nurses. Nurs Womens Health.

[8] Mei JY, Afshar Y, Gregory KD, Kilpatrick SJ, Esakoff TF (2016). Birth plans: what matters for birth experience satisfaction. Birth.

[9] Ayers S, Pickering AD (2001). Do women get post-traumatic stress disorder as a result of childbirth? A prospective study of incidence. Birth.

[10] Wijma K, Söderquist J, Wijma B (1997). Posttraumatic stress disorder after childbirth: a cross sectional study. J Anxiety Disord.

[11] Czarnocka J, Slade P (2000). Prevalence and predictors of post-traumatic stress symptoms following childbirth. Br J Clin Psychol.

[12] Righetti-Veltema M, Conne-Perréard E, Bousquet A, Manzano J (1998). Risk factors and predictive signs of postpartum depression. J Affect Disord.

[13] Ryding EL, Wijma K, Wijma B (1998). Postpartum counselling after an emergency cesarean. Clin Psychol Psychother.

[14] Priest SR, Henderson J, Evans SF, Hagan R (2003). Stress debriefing after childbirth: a randomised controlled trial. Med J Aust.

[15] Sepahvand H, Mokhtari Hashtjini M, Salesi M, Sahraei H, Pirzad JG. Prevalence of post-traumatic stress disorder (PTSD) in Iranian population following disasters and wars: a systematic review and meta-analysis. Iran J Psychiatry Behav Sci. 2019;13(1):e66124.

[16] Waldenström U (1999). Experience of labor and birth in 1111 women. J Psychosom Res.

[17] Yam EA, Grossman AA, Goldman LA, García SG (2007). Introducing birth plans in Mexico: an exploratory study in a hospital serving low-income Mexicans. Birth.

[18] Hidalgo-Lopezosa P, Hidalgo-Maestre M, Rodríguez-Borrego MA. Birth plan compliance and its relation to maternal and neonatal outcomes. Rev Lat Am Enfermagem. 2017;25. 10.1590/1518-8345.2007.2953 PMC5738855 29236838

[19] Azami-Aghdash S, Ghojazadeh M, Dehdilani N, Mohammadi M (2014). Prevalence and causes of cesarean section in Iran: systematic review and meta-analysis. Iran J Public Health.

[20] Mehri N, Messkoub M, Kunkel S. Trends, determinants and the implications of population aging in Iran. Working Paper No. 646. International Institute of Social Studies; August 2019. Avaiable at: file:///C:/Users/sony/Downloads/wp646.pdf. Accessed 20 July 2020.

[21] Ghanbari-Homayi S, Fardiazar Z, Meedya S, Mohammad-Alizadeh-Charandabi S, Asghari-Jafarabadi M, Mohammadi E (2019). Predictors of traumatic birth experience among a group of Iranian primipara women: a cross sectional study. BMC Pregnancy Childbirth.

[22] Gibbins J, Thomson AM (2001). Women's expectations and experiences of childbirth. Midwifery.

[23] Ghanbari-Homayi S, Dencker A, Fardiazar Z, Jafarabadi MA, Mohammad-Alizadeh-Charandabi S, Meedya S (2019). Validation of the Iranian version of the childbirth experience questionnaire 2.0. BMC Pregnancy Childbirth.

[24] CoxJL HJ, Sagovsky R (1987). Detection of postnatal depression: development of the 10-item Edinburgh postnatal depression scale. Br J Psychiatry.

[25] Montazeri A, Torkan B, Omidvari S (2007). The Edinburgh postnatal depression scale (EPDS): translation and validation study of the Iranian version. BMC psychiatry.

[26] Serçekuş P, İsbir GG, İnci FH. Reliability and validity of the delivery fear scale, Dokuz Eylül Üniv Hemşirelik Fakül Elektronik Derg. 2017;10(4):179–85.

[27] Toohill J, Fenwick J, Gamble J, Creedy DK, Buist A, Turkstra E (2014). A randomized controlled trial of a psycho-education intervention by midwives in reducing childbirth fear in pregnant women. Birth.

[28] Mortazavi F (2017). Validity and reliability of the Farsi version of Wijma delivery expectancy questionnaire: an exploratory and confirmatory factor analysis. Electron Physician.

[29] Kitzinger S (1999). Birth plans: how are they being used?. Br J Midwifery.

[30] Mirzamani M, Mohammadi M, Besharat M (2006). Application of the PTSD symptoms scale (PSS) for Iranian PTSD patients. Med J Islam Repub Iran.

[31] Lavender T, Tsekiri E, Baker L (2008). Recording labour: a national survey of partogram use. Br J Midwifery.

[32] Hsieh H-F, Shannon SE (2005). Three approaches to qualitative content analysis. Qual Health Res.

[33] Mayring P (2004). Qualitative content analysis. Companion Qual Res.

[34] Graneheim UH, Lundman B (2004). Qualitative content analysis in nursing research: concepts, procedures and measures to achieve trustworthiness. Nurse Educ Today.

[35] Berg M, Lundgren I, Lindmark G (2003). Childbirth experience in women at high risk: is it improved by use of a birth plan?. J Perinat Educ.

[36] Pinar G, Avsar F, Aslantekin F (2018). Evaluation of the impact of childbirth education classes in Turkey on adaptation to pregnancy process, concerns about birth, rate of vaginal birth, and adaptation to maternity: a case-control study. CNR.

[37] Epstein RM, Street RL. The values and value of patient-centered care. Annals Family Med. 2011;9(2):100–3. 10.1370/afm.1239 PMC3056855 21403134

[38] Tillett J (2009). Decision making by women during the process of labor. J Perinat Neonatal Nurs.

[39] Elwyn G, Dehlendorf C, Epstein RM, Marrin K, White J, Frosch DL (2014). Shared decision making and motivational interviewing: achieving patient-centered care across the spectrum of health care problems. Ann Fam Med.

